# Effect of Electroacupuncture on Rat Ischemic Brain Injury: Importance of Stimulation Duration

**DOI:** 10.1155/2013/878521

**Published:** 2013-05-13

**Authors:** Fei Zhou, Jingchun Guo, Jieshi Cheng, Gencheng Wu, Ying Xia

**Affiliations:** ^1^Shanghai Research Center for Acupuncture and Meridians, Shanghai 201203, China; ^2^Gongli Hospital, Pudong New District, Shanghai 200135, China; ^3^Shanghai Medical College of Fudan University, Shanghai 200032, China; ^4^The University of Texas Medical School at Houston, Houston, TX 77030, USA; ^5^Yale University School of Medicine, New Haven, CT 06520, USA

## Abstract

We explored the optimal duration of electroacupuncture (EA) stimulation for protecting the brain against ischemic injury. The experiments were carried out in rats exposed to right middle cerebral artery occlusion (MCAO) for 60 min followed by 24-hr reperfusion. EA was delivered to “Shuigou” (Du 26) and “Baihui” (Du 20) acupoints with sparse-dense wave (5/20 Hz) at 1.0 mA for 5, 15, 30, and 45 min, respectively. The results showed that 30 min EA, starting at 5 minutes after the onset of MCAO (EA during MCAO) or 5 minutes after reperfusion (EA after MCAO), significantly reduced ischemic infarct volume, attenuated neurological deficits, and decreased death rate with a larger reduction of the ischemic infarction in the former group. Also in the group of EA during MCAO, this protective benefit was positively proportional to the increase in the period of stimulation, that is, increased protection in response to EA from 5- to 30-min stimulation. In all groups, EA induced a significant increase in cerebral blood flow and promoted blood flow recovery after reperfusion, and both blood flow volume and blood cell velocity returned to the preischemia level in a short period of time. Surprisingly, EA for 45 min did not show reduction in the neurological deficits or the infarct volume and instead demonstrated an increase in death rate in this group. Although EA for 45 min still increased the blood flow during MCAO, it led to a worsening of perfusion after reperfusion compared to the group subjected only to ischemia. The neuroprotection induced by an “optimal” period (30 min) of EA was completely blocked by Naltrindole, a **δ**-opioid receptor (DOR) antagonist (10 mg/kg, i.v.). These findings suggest that earlier EA stimulation leads to better outcomes, and that EA-induced neuroprotection against ischemia depends on an optimal EA-duration via multiple pathways including DOR signaling, while “over-length” stimulation exacerbates the ischemic injury.

## 1. Introduction

Ischemic/hypoxic brain injury, such as stroke, leads to serious and complex pathophysiological changes affecting multiple levels of the brain, and sports a high global mortality rate as the leading cause of neurological disability [1–9]. In spite of extensive research conducted in the past several decades, limited therapeutic options are available against ischemic/hypoxic brain injury till date. The vast physical, emotional, and financial tolls that stroke inflicts upon patients and their families imposes a daunting and continuous challenge to the medical community [5–9]. Previous studies at our laboratory and at those of others have shown that electroacupuncture (EA), at appropriate acupoints with suitable stimulation parameters, significantly attenuates neurological deficits, infarct volume and mortality in animal models exposed to ischemic insults [10–17], which may potentially shed a new light on developing a better modality for ischemic brain injury. Indeed, there is substantial clinical evidence demonstrating beneficial effects of acupuncture on stroke patients [18–21]. 

Because of inadequate control, poor methodological quality and small samples seen in previous studies, the efficacy of acupuncture on hypoxic/ischemic injury is still unproven in clinical settings. In fact, major controversies exist with regards to the effectiveness of acupuncture in stroke patients [22–24]. These may, at least partially, be attributed to the varied approaches adopted in different studies, and since acupuncture induces complex changes at multiple levels in the central nervous system, the outcomes also differ with varying acupunctural methods [25–30]. Therefore, it is extremely important to define what optimal parameters are required for acupuncture treatment in ischemic/hypoxic brain injury.

Towards this goal, our serial studies have established that EA treatment for acute stroke in experimental animals is critically dependent upon specific acupoints along with stimulation currents of specific intensities and frequencies [11–15, 17]. Since the timing and duration of medical treatment has a huge impact on patients with acute stroke [7–9], we conducted this work to address two fundamental issues, that is, (1) when should EA be applied to induce optimal protection, during or after ischemia?, and (2) what duration of EA application is optimal to induce maximal beneficial effect on the ischemic brain?

## 2. Materials and Methods

### 2.1. Animals

Adult male Sprague-Dawley rats (240–270 g) were used for the experiments. All animals were purchased with permission from the Experimental Animal Center for the Shanghai Chinese Academy of Science. The animals were housed at an ambient temperature of 24 ± 1°C and were provided with free access to food and water. All surgical procedures were approved by the Animals Care and Use Committees of Fudan University Shanghai Medical College, Shanghai, China, and were performed under anesthesia (chlorate hydrate, 400 mg/kg, i.p.).

### 2.2. Cerebral Blood Flow Monitoring

The cerebral blood flow was measured using laser-Doppler perfusion monitor (LDPM, PeriFlux5000, Perimed, Sweden). The major steps of the procedure were as follows: first, a small hole was drilled in the right parietal bone at a point 1.5 mm posterior to the bregma and 5 mm lateral to the sagittal suture, as previously described [14, 17, 31]. The superficial micro-vessels of cerebral pia mater were accessed using a laser Doppler probe (0.45 mm diameter) inserted into the hole at a depth of 2 mm and fixed to the skull bone. The probe was used to measure the blood perfusion to the cortex to record the cerebral blood flow. Continuous monitoring of cerebral blood flow was performed beginning 5 minutes prior to the induction of cerebral ischemia until at least 15 minutes after reperfusion. As per the manufacturer's guidelines, the measured change in perfusion values was dependent on the number of blood cells present and their velocity in the area illuminated by the tip of the probe. The changes in the cerebral blood flow of a localized area, recorded as a measure of the dynamic changes in Perfusion Units (PU), concentration of the moving blood cells (CMBC), and the velocity of blood cells (Velocity), were constantly monitored in real-time using LDPM. PU is an index of cerebral blood flow and is expressed as the product of the number of moving blood cells and their relative velocities. 

### 2.3. Method for Inducing Cerebral Ischemia

We followed the methods described by Longa et al. [32] that are also detailed in our previous publications [14, 17] for creating a focal cerebral ischemia model by middle cerebral artery occlusion (MCAO). Animals were taken under anesthesia to surgically expose their right common carotid, external carotid and internal carotid arteries. After ligation of the distal end of the right external carotid artery, it was incised and a 4–0 monofilament nylon suture (30 mm length, 0.18 mm diameter with a 0.24 mm diameter round tip, MONOSOF, SN-1699G, USA) was introduced to the right external carotid artery and further into the right internal carotid artery for ~20 mm up till the origin of the right middle cerebral artery. The blood flow to the right middle cerebral artery was then occluded at this level.

We constantly monitored the blood flow in all animals to ensure that a uniform cerebral ischemia level was maintained across all groups in a standard fashion. The blood flow was controlled by adjusting the suture in the artery for the induction of ischemia. The ischemic rats that showed a stable drop of ~85% in PU compared to the baseline level (before MCAO), that is, reaching a level of ~15% of the baseline PU, were used for further experimentation. The PU was kept at a low level with minor fluctuations during the entire ischemic period without any significant change except during EA application. After the occlusion, reperfusion of the ischemic area was allowed by withdrawing the suture from the right external carotid artery. Cortical blood flow changes were monitored in all animals beginning 5 minutes prior to the induction of cerebral ischemia until at least 15 minutes after reperfusion. This duration was inclusive of the entire length of MCAO and of EA as well.

Body temperature was maintained at 36.5°C ± 0.5°C throughout the surgical procedures till the animal recovered from anesthesia. After the reperfusion, the animals were housed for 24 hours at an ambient temperature of 24 ± 1°C. The same surgical procedures were performed on the animals in the sham-operation group, excluding MCAO. 

### 2.4. Electroacupuncture Methods

WHO standards were followed for the name and localization of acupoints [33, 34]. “Shuigou (Du 26)” and “Baihui (Du 20)” are acupoints located on the head and face. Shuigou (Du 26) is located at a point on the midline of the upper lip 2/3rds from the mouth to the nose. Baihui (Du 20) is located at a point on the midline of the head, approximately midway on the line connecting the apices of both auricles. 

To obtain optimal EA effect that induces maximum protection against cerebral ischemia, we applied EA at “Shuigou” (Du 26) and “Baihui” (Du 20) with 5/20 Hz sparse-dense frequency at a constant intensity of ~1.0 mA in all EA groups, beginning at 5 min after the onset of MCAO or 5 min after MCAO. The selection of the acupoints and stimulation parameters was based on the results of our serial studies in the past [14, 17]. 

In determination of effects of different EA periods on cerebral ischemia, EA was applied using varying lasting-durations, that is, beginning at 5 min after the onset of MCAO and continued up to 5 minutes, 15 minutes, 30 minutes, and 45 minutes, respectively. Five different groups were studied in the present study with the numbers in each group ranging from 16 to 45: MCAO only (Ischemia, *n* = 45), MCAO plus EA for 5 min (EA-5 min, *n* = 18), MCAO plus EA for 15 min (EA-15 min, *n* = 16), MCAO plus EA for 30 min (EA-30 min, *n* = 30), and MCAO plus EA for 45 min (EA-45 min, *n* = 30). 

EA was delivered through stainless steel filiform needles (15 mm in length and 0.3 mm in diameter, Suzhou Medical Apparatus Limit Co., China) by an EA apparatus (Model G-6805-II, Shanghai Medical Instruments High-Tech Co., China). The needle on Du 26 is inserted 1 mm deep, vertical to the plane of the skin. At Du 20, the needle is inserted obliquely and to a depth of 2 mm [14, 17, 25, 33, 34]. The intensity and frequency of the output waves with a negative-going pulse on the posterior border (pulse width = 0.5 ms ± 0.1 ms; component of direct current = Zero) were monitored on a general oscillograph (Model XJ4210A, Shanghai XinJian Instrument and Equipment Co., China).

### 2.5. *δ*-Opioid Receptors Blockade

The rats were randomly assigned to one of the four experimental groups: (1) Ischemia alone (*n* = 7); (2) Ischemia + EA at “Shuigou” (DU 26) and “Baihui” (DU 20) (*n* = 8); (3) Ischemia + Naltrindole (*n* = 8), in which rats were administered Naltrindole (10 mg/kg i.v.) 5 min before the onset of MCAO; and (4) Ischemia + EA + Naltrindole (*n* = 10), in which the animals were treated with both EA and Naltrindole. EA was started 5 min after ischemia and continued to 35 min after ischemia in this set of experiments.

### 2.6. Mortality and Neurological Deficits Monitoring

Some ischemic rats died between 2 and 20 hrs after reperfusion. The death rates were reported for this period based on the number of dead rats and the total number of the rats allocated to the given group. A myriad of factors could be implicated as the cause of death, as for example hemorrhage. However, investigating the precise cause of death was not our aim in this work. 

Assessments on neurological deficits were made on animals in all groups. The rats that died within 24 h after MCAO were excluded. Assessments were made 24 h after reperfusion just before the sampling of brain tissue was done. The assessments on neurological deficits were blinded, that is, the person evaluating and scoring the neurological deficits based on pre-set criteria had no prior knowledge on the groups and treatments. The degree of neurological deficits was graded from 0–7 grades [14, 17]. The criteria were set as follows: Grade 0—“normal”, symmetrical movement without any abnormal sign; Grade 1—incomplete stretch of the left anterior limb when the tail was lifted up; Grade 2—doddery crawl along with the signs of Grade 1; Grade 3—kept the left anterior limb close to the breast when the tail was lifted up; Grade 4—left turn when crawling; Grade 5—left anterior claw pushed backward along with the signs of Grade 4; Grade 6—repeated rotational motion with an immotile posterior left limb; and Grade 7—left recumbent position because of body supporting incapability.

### 2.7. Cerebral Infarct Measurement

After the assessment of neurological deficits, animals were sacrificed under anesthesia and samples of brain were taken. The brain slices were prepared as 2.0 mm sections (*n* = 12–16) and were incubated in a solution of triphenyltetrazolium chloride (TTC, 20 g/L) for 30 minutes at 37°C before being transferred into paraformaldehyde solution (40 g/L) for fixing the infarcted area. The infarct region appeared white or pale while the “normal” tissue appeared red [35, 36]. Pictures were taken of the brain slices with a digital camera and the volume of infarct was analyzed using a computer-assisted image system with ACT-2U software (Nikon). Relative infarct ratio was calculated using the following equation [14, 17, 31]: (2 ∗ left hemisphere area (non-ischemic side) − non-infarct area of whole brain slices)/(2 ∗ left hemisphere areas) ∗ 100%. This equation excludes the factors that could result in an inaccurate calculation of the infarct volume (such as edema). 

### 2.8. Data Analysis

The cerebral blood flow was calculated from PU, CMBC, and velocity measurements. All individual measurements were compared to the baseline values, before MCAO (control level), in each animal. The grouped values were compared between different groups. An average grade of neurological deficits per group was used to make comparisons between groups. Cerebral infarct volume was expressed as a percent fraction of the entire cerebral volume. 

All data is presented as mean ± SD and subjected to statistical analysis. The rate of death was compared using the Chi-square test. All other data was subjected to Analysis of variance (ANOVA), *t*-test, Rank-Sum test, and/or Chi-square test. The changes were considered to be significant if the *P* value was less than 0.05. 

## 3. Results

### 3.1. Different Effects Induced by EA Starting after the Onset of MCAO versus Post-MCAO

**Figure 1:**
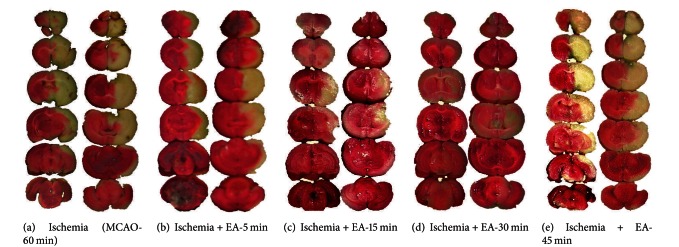
EA-induced changes in cerebral infarct size in a time-dependent manner. The brain slices were subjected to TTC staining and the ischemic infarct volume was quantified by a computerized image system. The slices on the *right *of each column show the backside of the *left *slices. Note that the infarct region (pale-white portion) was mainly located in the striatum and the frontoparietal cortex in the right hemisphere. The MCAO-induced infarction (a) was significantly reduced by EA at Du 20 and Du 26 acupoints for 5 min (b), 15 min (c), and 30 min (d). In contrast, EA for 45 min (e) enabled no protection against the cerebral infarction.

In the group of Ischemia (*n* = 45), with 1 hr MCAO and 24 hr reperfusion, about 18% (8/45) of the rats died within 2–20 hours after the onset of reperfusion. At 24 hours of reperfusion, the living rats displayed serious neurological deficits (Grade 6.0 ± 0.5, *n* = 37). TTC staining showed that the volume of cerebral infarct was about one third of the whole brain (32.8% ± 3.7%, *n* = 16) (Table 1, Figure 1(a)). The infarct areas were mainly localized in the frontoparietal lobes of the cortex and striatum in the right hemisphere (ischemic side, Figure 1(a)).

EA starting at 5 min after the onset of MCAO induced a marked protection against cerebral ischemia, leading to a significant reduction of infarct volume, neurological deficits, and death rate (Table 1, Figures 1(b)–1(d)). EA starting at 5 min after MCAO induced a similar outcome in the ischemic rats (refer to Figure 6). However, it seemed that EA induced a larger reduction of the infarct volume in the former than the later group. EA after the onset of MCAO reduced the infarct volume by ~85% (Table 1 and Figure 1), while EA after MCAO, by ~45% (refer to Figure 6). These results suggest that early institution of EA stimulation induces better protection against cerebral ischemic injury. 

### 3.2. Increased EA Protection with Increased Periods of the Stimulation from 5 to 30 mins

In the group of MCAO plus EA for 5 min (EA-5 min, *n* = 18), except for 2 rats that died at 5 and 15 hours after the onset of reperfusion (11%, 2/18, *P* < 0.01 versus Ischemia), the degree of average neurological deficits in the living ischemic rats was slightly improved (Grade 5.0 ± 0.5, *n* = 16, *P* < 0.05 versus Ischemia). The infarct volume was slightly reduced (25.6% + 5.3%, *n* = 12, *P* < 0.05 versus Ischemia) (Table 1 and Figure 1(b)). In the group of MCAO plus EA for 15 min (EA-15 min, *n* = 16), only one rat died at ~3 hours after the onset of reperfusion (6%, 1/16, *P* < 0.01 versus Ischemia), with a greater improvement in average neurological deficits (Grade 3.0 ± 0.5, *n* = 15, *P* < 0.01 versus Ischemia) along with a significant reduction in infarct volume (15.4% ± 4.2%, *n* = 12, *P* < 0.01 versus Ischemia) (Table 1 and Figure 1(c)). In the group of MCAO plus EA for 30 min (EA-30 min, *n* = 30), the neurological deficits were greatly attenuated (Grade 1.0 ± 0.5, *n* = 28, *P* < 0.01 versus Ischemia) and a significant decrease in death rate (7%, 2/30, *P* < 0.01 versus Ischemia) was noted. The infarct volume was reduced by 85% (4.9% ± 1.2%, *n* = 12, *P* < 0.01 versus Ischemia) (Table 1 and Figure 1(d)). In comparison to the groups of EA for 5 min and EA for 15 min, EA for 30 min induced more beneficial effects in all aspects including neurological deficits, ischemic infarct and death rate (Table 1, Figures 1(b)–1(d)). These results suggest that the EA protection is dependent on an appropriate EA duration. 

### 3.3. Exacerbation of Ischemic Injury by “Over-Length” Stimulation of EA

We investigated to see if the EA protection would be further enhanced by a longer duration of EA stimulation. Therefore, we assigned a few rats to a new group with MCAO plus EA for 45 minutes (EA-45 min, *n* = 30). To our surprise, EA for 45 min significantly increased the mortality in this group of ischemic rats. More than half of the animals in this group (60%, 18/30) died within 0.5 to 10 hours after the onset of reperfusion. All of the dying rats manifested symptoms such as convulsions, tumbling, piloerection, and perspiration (wet feathers) and other abnormalities. When compared the Ischemia group, the death rate increased by 3 folds (60%, 18 out of 30, *P* < 0.01 versus Ischemia) in this group. Although the remaining 12 living rats survived for 24 hours after the reperfusion, they suffered from severe neurological deficits (Grade 7) and were even worse than the group with only Ischemia (Grade 6.0 + 0.5 with range of 5 ~ 7, *P* < 0.05). In terms of the infarct volume, EA for 45 min did not reduce the infarct volume at all (34.3% ± 2.4%, *n* = 12, *P* > 0.05 versus Ischemia). (Table 1, Figure 1(e)). These results suggest that an increased duration of EA stimulation application further exacerbates the ischemic insult, instead of conferring any protection. 

### 3.4. EA-Induced Increase in Cerebral Blood Flow during MCAO

**Figure 2:**
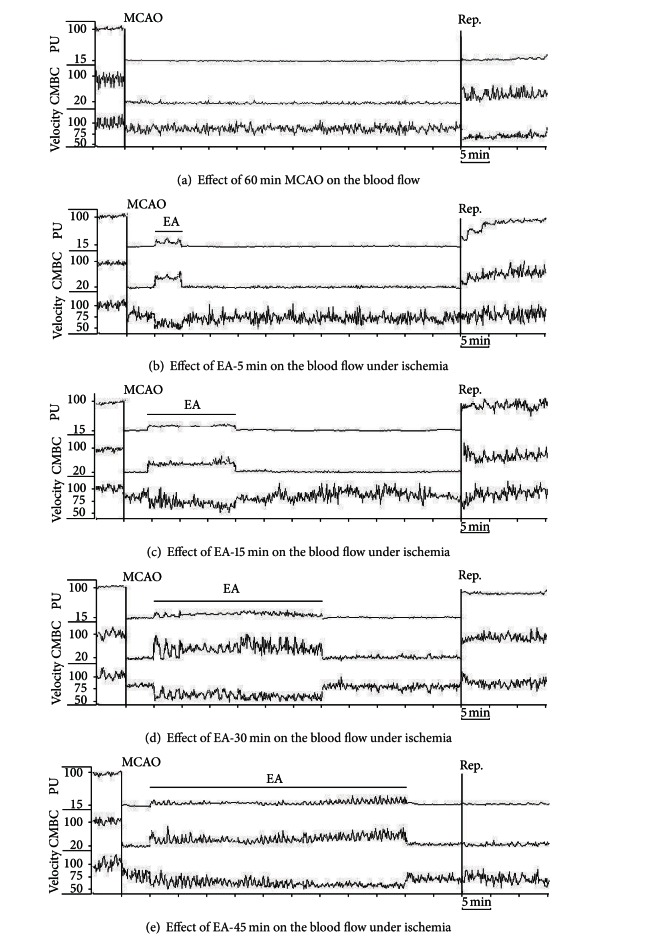
Representative trace recordings of the blood flow in the rats with or without EA. Blood Perfusion (PU), Concentration of Moving Blood Cells (CMBC), and Velocity of Blood Cells (Velocity) were measured in the ischemic rats by a Laser Doppler Perfusion Monitor system. (a) Effect of MCAO-60 min on CBF during ischemia and after reperfusion in Ischemia group. (b) Effect of EA for 5 min at acupoints of Du 20 and Du 26 on CBF. (c) Effect of EA for 15 min on CBF. (d) Effect of EA for 30 min on CBF. (e) Effect of EA for 45 min on CBF. Note that the PU and CMBC decreased immediately after MCAO and the blood flow was maintained at a low level with small fluctuations during the entire period of MCAO with a slight decrease in the velocity. After the onset of reperfusion, PU and CMBC increased with a further decrease in the velocity. EA induced an isochronous increase in PU and CMBC with a decrease in velocity during MCAO. After reperfusion, PU, CMBC, and velocity all increased rapidly and reached the baseline values within 15 min after reperfusion onset in groups of EA for 5–30 min. EA for 45 min, however, induced a greater decrease in PU, CMBC, and velocity than the changes observed in the Ischemia group after reperfusion.

**Figure 3:**
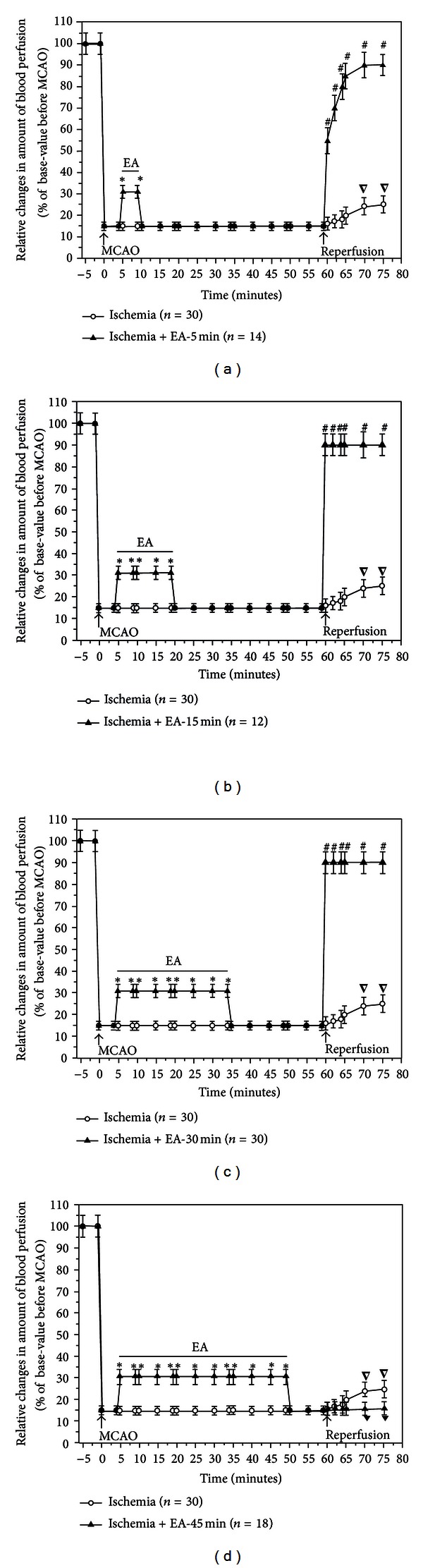
Statistical summary of EA effects on PU in the ischemic brain. *P < 0.01, Ischemia + EA versus Ischemia group during MCAO; ∇*P* < 0.05, under reperfusion versus during MCAO in the Ischemia group; ^#^ *P* < 0.01, Ischemia + EA versus Ischemia group under reperfusion; ^*▼*^ *P* < 0.05, Ischemia + EA versus Ischemia group under reperfusion. Note that MCAO sharply decreased PU to ~15% of the basal value (pre-MCAO), while EA for 5–45 min significantly increased the blood flow to ~30% of the baseline. Within 10–15 minutes after reperfusion, PU gradually increased to ~25% of the baseline (pre-MCAO) in the Ischemia group, whereas it rapidly increased to about 90% of the baseline in the EA for 5–30 minutes groups. In sharp contrast, PU was still at the MCAO level, that is, ~15% of the basal value, during the same period after reperfusion in the group of EA for 45 minutes.

**Figure 4:**
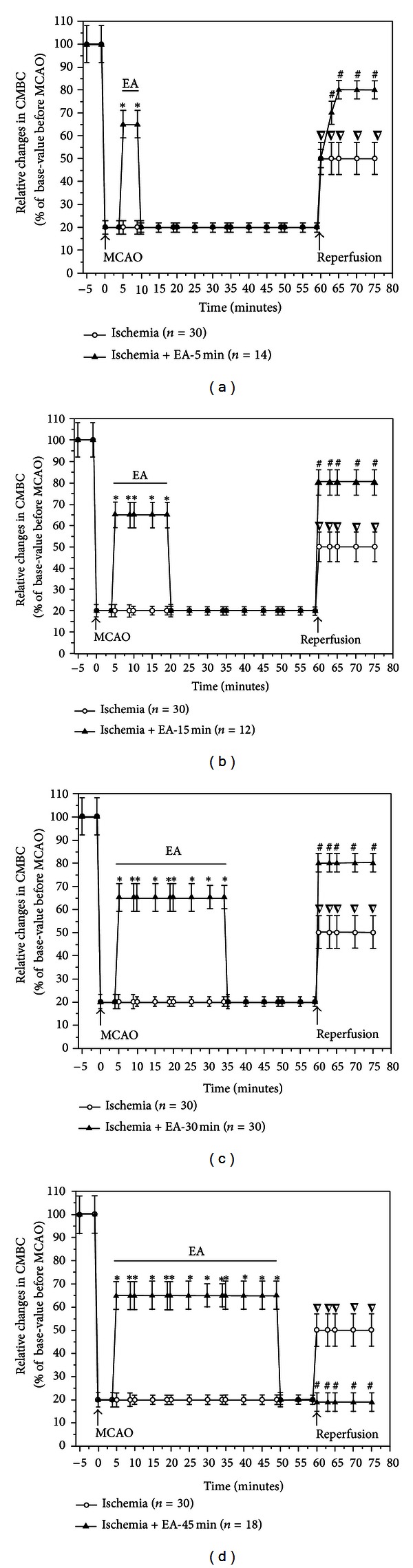
Statistical summary of EA effects on concentration of Moving Blood Cells.*P < 0.01, Ischemia + EA versus Ischemia group during MCAO; ∇*P* < 0.01, under reperfusion versus during MCAO in Ischemia group; ^#^ *P* < 0.01, Ischemia + EA versus Ischemia group under reperfusion. Note that the MCAO greatly reduced the concentration of Moving Blood Cells (CMBC) to ~20% of the baseline and CMBC quickly increased to ~50% of the baseline after reperfusion. EA for 5–45 minutes significantly increased CMBC during MCAO to ~65% of the baseline. Within 15 minutes after reperfusion, CMBC further increased to ~80% of the baseline in the EA for 5–30 minutes groups, but not in the EA for 45 minutes group with its level being similar to that during MCAO.

**Figure 5:**
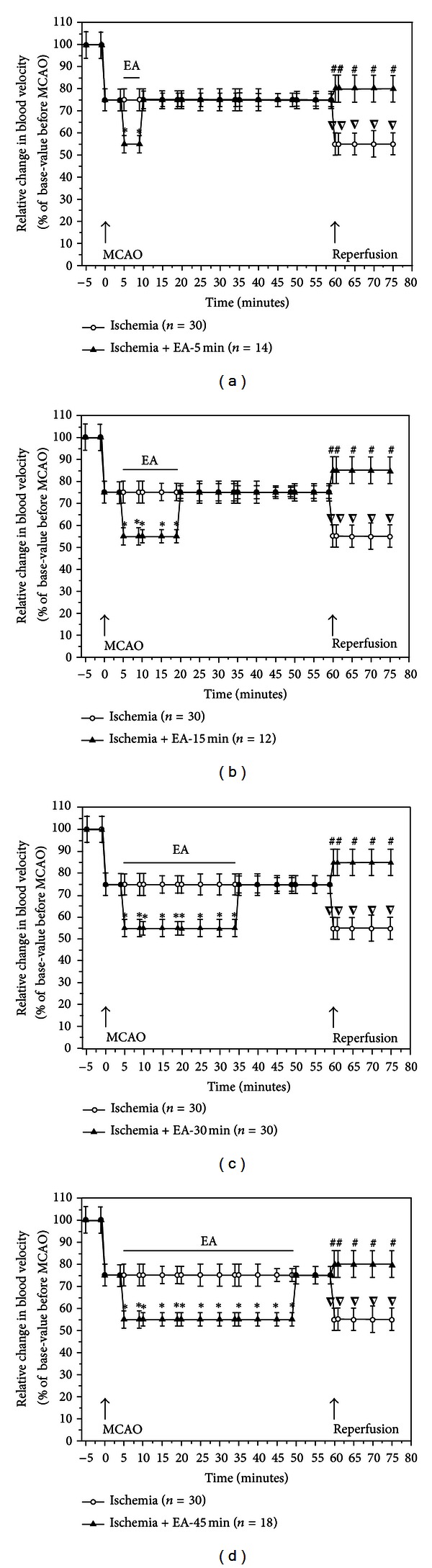
Statistical summary of EA effects on the velocity of blood cells.*P < 0.05, Ischemia + EA versus Ischemia group during MCAO; ∇*P* < 0.05, under reperfusion versus during MCAO in Ischemia group; ^#^ *P* < 0.05, Ischemia + EA versus Ischemia group under reperfusion. Note that MCAO reduced the velocity of the blood cells to ~75% of the baseline, which was further decreased to ~55% of the baseline after reperfusion. In all EA groups, EA during MCAO immediately decreased the velocity to ~55% of the baseline, and induced a significant increase in it after reperfusion. In contrast to changes in PU and CMBC, the velocity increased to >80% after reperfusion in all EA groups including EA-45 min.

Using a Laser-Doppler Perfusion Monitor, we continuously monitored the real time changes in cerebral blood flow in all the groups beginning at 5 min prior to MCAO till 15 min after the onset of reperfusion (suture withdrawal), and compared differences in the cerebral blood flow at the pre-MCAO level, during MCAO with/without EA, and early stages of reperfusion between various groups (Figures 2, 3, 4, and 5). 

 Consistent with our previous studies [14, 17], after the insertion of a nylon suture into the right middle cerebral artery, the blood perfusion (PU) to the monitored cortex immediately decreased by ~85% (i.e., reaching the level at ~15% of the pre-MCAO level). During the entire period of MCAO, the local blood flow was maintained at this low level with a minor fluctuation. CMBC dropped by ~80% of the base-value (i.e., reaching a level at ~20% of the pre-MCAO level) with a slight deceleration of blood cell velocity (reduced by ~25% of the pre-MCAO level). These changes indicated that MCAO induced a greater reduction in the blood volume as compared to the velocity of blood cells (Figures 2(a) and 3–5).

In all the EA groups, EA induced an instant and significant increase in the blood flow of the ischemic brain. The blood flow changed isochronously with the current impulse in response to EA stimulation (Figures 2(b)–2(e) and 3–5). EA induced an increase in PU to ~30% of the control (*P* < 0.01 versus MACO without EA) (Figure 3) and CMBC to over 65% of the control (*P* < 0.01 versus MCAO without EA) (Figure 4) with a simultaneous decrease in the blood cell velocity to ~55% of the control (*P* < 0.05 versus MCAO without EA) (Figure 5). These results suggest that both “appropriate” and “over” periods of EA stimulation increase the blood flow during MCAO.

### 3.5. Differential Recovery of the Cerebral Blood Flow after Reperfusion among EA Groups

In the Ischemia group (*n* = 30), following the withdrawal of the nylon suture after 1 h MCAO, PU gradually increased to ~25% of the control level (pre-MCAO) (*P* < 0.05 versus during MCAO) and CMBC increased to ~50% of the control (*P* < 0.01 versus during MCAO) with the velocity of blood cells being reduced from ~75% to ~55% (*P* < 0.05, versus during MCAO) in the first 15 minutes after the blood reperfusion (Figures 2(a) and 3–5). 

EA changed the pattern of the blood reperfusion. There was, however, a significant difference among different EA groups, which was evident in the early stage of reperfusion, for instance, during the first 15 minutes after MCAO. In the groups of EA-5 min (*n* = 14), EA-15 min (*n* = 12), and EA-30 min (*n* = 30), blood perfusion (PU) increased to over 90% of the base-value (preischemia) (Figure 3, *P* < 0.01 versus the ischemia group under reperfusion), CMBC recovered to >80% of the preischemia level (Figure 4, *P* < 0.01 versus the ischemia group under reperfusion) and the Velocity increased to >80% of the control level (Figure 5, *P* < 0.05 versus the ischemia group under reperfusion) within 15 minutes after reperfusion. 

In sharp contrast, the EA-45 min group (*n* = 18) showed a totally different pattern. For example, PU maintained at a similar level as that during MCAO (~15%), that is, after reperfusion, its level was even lower than those of Ischemia alone (~25%, *P* < 0.05 versus that during reperfusion in the group of Ischemia plus EA for 45 minutes) (Figure 3). Similarly, CMBC still maintained at a low level (~20%) after reperfusion and the level was even lower than that (50%) of the Ischemia group during reperfusion (*P* < 0.01, Figure 4). These findings suggest that EA for 30 minutes or less increases the blood flow during MCAO and promotes the recovery of the blood flow during reperfusion, while an “over-length” (45 min) stimulation, though increasing the blood flow during MCAO, retards the recovery of the blood flow to the brain after MCAO (Figures 2(e) and 3–5).

### 3.6. DOR Antagonist Attenuated EA-Induced Protection against Ischemic Infarction

**Figure 6:**
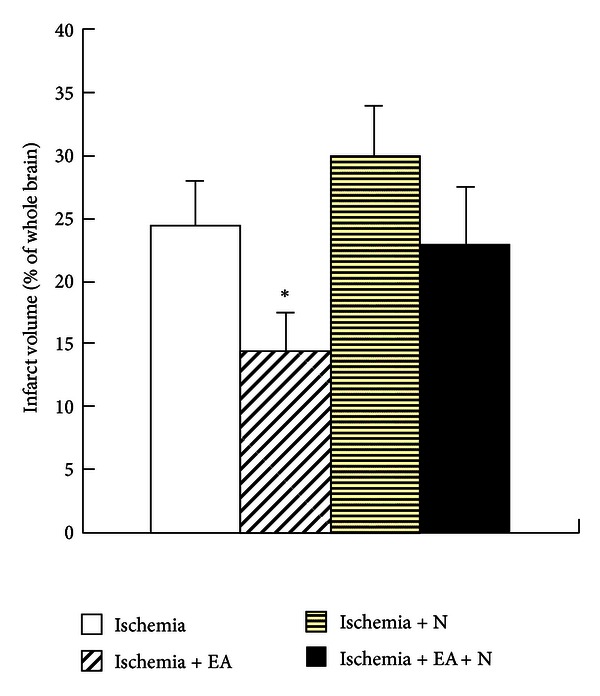
Effect of Naltrindole on EA-induced protection against ischemic infarct size. Quantitative volumes of the ischemic infarct were determined based on the percentage of the whole brain volume. Note that the ischemia-induced infarct was reduced by the EA stimulation (*P < 0.05 versus ischemia group), which was almost completely reversed after treatment with Naltrindole (N, 10 mg/kg, i.v.) (*P < 0.05 versus Ischemia + EA + Naltrindole).

We previously found that microinjection of DOR antagonist into the lateral cerebral ventricle largely attenuated the EA protection against cerebral ischemic injury [10]. To further reaffirm this observation, we applied Naltrindole to the ischemic rats and determined its effect on the EA-induced neuroprotection. The results showed that in the ischemia group (*n* = 7) the infarct size reduced by ~40% (from 24.8% ± 4.1% to 14.7% ± 3.4%, *P* < 0.05) following treatment with EA beginning immediately after MCAO (*n* = 8). Although Naltrindole (10 mg/kg, i.v.) application did tend to increase the infarct volume, the change was not statistically significant (30.4% ± 4.3%, *n* = 8, *P* > 0.05 versus Ischemia alone). However, it completely abolished the EA-induced protection against ischemic infarction (23.6% ± 5.2%, *n* = 10, *P* > 0.05 versus Ischemia + EA) (Figure 6). These results indicate that *δ*-opioid receptor plays a very important role in the EA-induced protection against ischemic injury.

## 4. Discussion

This is the first study to systematically define optimal duration of EA stimulation to induce beneficial effects against ischemic infarction, neurological deficits and mortality. Our results show that EA given for 5–30 minutes significantly reduces ischemic infarct volume and decreases neurological deficits and mortality rate. On the other hand, EA given for 45 min did not show reduction in the neurological deficits or the infarct volume, and rather demonstrated an increase in death rate in this group. However, all groups showed an increase in blood flow after EA stimulation during the period of MCAO. These findings are consistent with our preliminary observations [37] and suggest that the EA protection against ischemic injury is dependent on the length of EA stimulation, and that an “over-length” stimulation can potentially add to the ischemic injury instead of protecting the brain from the injury.

This observation was beyond our expectations. Since the primary cause of ischemic brain injury is insufficient blood supply to the brain, increased blood flow to the brain should reduce the cerebral ischemic injury. Our previous studies concluded that EA for 30 minutes increases the blood flow during MCAO and reduces ischemic insult to the brain [11, 12, 14, 17]. In extension, we speculated that an extended period of EA stimulation would produce better outcomes. Apparently, this was not the case as we observed opposite results; that is, a prolonged stimulation of EA potentiates, rather than protects against, cerebral damage during ischemic injury to brain. Interestingly, Dr. Wang et al. [38] also observed a similar phenomenon in their studies on EA-induced hypoalgesia in healthy volunteers. The subjects were randomized to receive different durations (0 min, 20 min, 30 min, or 40 min) of asynchronous EA stimulations and then subjected to the test of hypoalgesia using a human experimental cold thermal pain threshold model. They found that 30 min of asynchronous EA stimulation resulted in the most significant hypoalgesic effect compared with 0, 20, or 40 min stimulations. Therefore, it seems that a 30 min period is the most optimal duration for EA-induced analgesia and brain protection against ischemic injury.

At present, the mechanism behind this phenomenon is not known. In this work, however, it is noteworthy that EA for 5–30 minutes significantly improves the reperfusion after MCAO and favors quick recovery of blood flow to the pre-ischemic level, in addition to increasing blood flow during MCAO. In sharp contrast, EA for 45 minutes does not promote the recovery of blood flow after MCAO at all, but to the contrary, further worsens it as shown in our present work. This phenomenon partially accounts for the difference in the outcomes between “optimal period” and “over-length” in the EA treatment although the underlying mechanism remains unknown. 

The fact that “over-length” of EA stimulation increases the blood flow under MCAO, but does not lead to any protection against ischemic injury strongly suggests that optimal EA stimulation induces a multi-level regulation in the brain via a complex mechanism. A single factor, such as, an increase in the blood flow under ischemic conditions, may not be enough to induce protection against ischemia. Appropriate stimulation is extremely important for maximal mobilization of various survival signals and activation of protective pathways in the ischemic brain. 

One of these pathways could involve DOR-mediated survival signaling. We have previously demonstrated that DOR is important in inducing neuroprotection [3, 6]. DOR activation attenuates hypoxic, ischemic, or excitatory injury in the neurons and the brain [1, 3, 6]. Evidence shows that both manual acupuncture and EA enhance the activity of the endogenous opioid system, including DOR, in the brain [26–29, 39]. Therefore, the DOR-mediated protection might play a very important role in the EA-induced protection against ischemic injury. Indeed, we first reported that microinjection of a DOR antagonist, Naltrindole, into the cerebral ventricle attenuated the EA-induced cerebral protection against ischemic infarction, suggesting DOR's involvement in the EA-induced protection against brain ischemia [10]. Furthermore, our studies and those of others suggest that EA significantly increases the density of DOR in the ischemic cortex and that DOR signaling could mediate the EA induced neuroprotection against ischemic injury [15, 39, 40]. In the present study, we observed that intravenous injection of Naltrindole almost completely blocked the EA-induced neuroprotection against ischemic injury. All of these observations consistently favor the critical role of DOR in EA-induced neuroprotection against cerebral ischemia. On the other hand, EA can activate additional neurotransmitter systems such as neurotrophic factors in the brain that enable further protection under ischemic conditions [13]. 

Thus, EA targets multiple mechanisms to achieve a protective effect against ischemic insults, including an increase in the cerebral blood flow under ischemia, an improved recovery of the blood flow after reperfusion, upregulation of the DOR pathway, and other survival signals. An optimal duration of EA stimulation can combine and direct all of these protective pathways against ischemic stress, while an over-stimulation of EA, despite increasing the blood flow during MCAO, can set this balance of combined protection off.

Moreover, our present results show that EA application during MCAO induces a stronger protection than EA after MCAO, suggesting that an early institution of EA treatment is required to achieve better neuroprotection against ischemic injury. This is similar to the goals of tPA treatment in terms of the importance of the therapeutic window [7, 8]. An early mobilization of protective power may prevent neurons from initiating the process of apoptosis/necrosis in the brain. “Time is brain,” which is also true in the case of EA treatment for ischemic brain injury.

In summary, our results suggest early EA treatment with optimal stimulation parameters for an appropriate period is critical to achieve a therapeutic effect for neuroprotection against ischemic insult. The previous controversies in the literature on EA treatment for stroke can be partially attributed to the differences in conditions of the patients and EA parameters. Further in-depth investigations into the “optimal” EA parameters and its underlying mechanisms can help improve clinical outcomes of patients with ischemic/hypoxic injury in the brain.

## Figures and Tables

**Table 1:** EA period-dependent protection against ischemic injury.

Groups	Neurological deficit	Infarct volume	Death rate
Ischemia	6.0 ± 0.5 (5 ~ 7) (*n* = 37)	32.8% ± 3.7% (*n* = 16)	18% (8 out of 45)
EA-5 min	5.0 ± 0.5 (4 ~ 6) (*n* = 16)^∗▲^	25.6% ± 5.3% (*n* = 12)^∗▲^	11% (2 out of 18)^∗∗▲^
EA-15 min	3.0 ± 0.5 (2 ~ 4) (*n* = 15)^∗∗▲^	15.4% ± 4.2% (*n* = 12)^∗∗▲^	6% (1 out of 16)^∗∗▲^
EA-30 min	1.0 ± 0.5 (0 ~ 2) (*n* = 28)^∗∗▲^	4.9% ± 1.2% (*n* = 12)^∗∗▲^	7% (2 out of 30)^∗∗▲^
EA-45 min	7.0 ± 0.0 (~7) (*n* = 12)*	34.3% ± 2.4% (*n* = 12)	60% (18 out of 30)**

*P < 0.05 versus Ischemia. **P < 0.01 versus Ischemia. ^▲^ *P* < 0.01 versus EA-45 min. Note that EA significantly reduced the infarct volume, neurological deficit, and death rate and this protective effect became better and better when the length of EA increased from 5 to 30 minutes. However, EA for 45 mins did not improve the neurological deficit and infarct volume and even increased the death rate.
